# Evidence for chikungunya and dengue transmission in Quelimane, Mozambique: Results from an investigation of a potential outbreak of chikungunya virus

**DOI:** 10.1371/journal.pone.0192110

**Published:** 2018-02-07

**Authors:** Vánio André Mugabe, Sadia Ali, Imelda Chelene, Vanessa Onofre Monteiro, Onélia Guiliche, Argentina Felisbela Muianga, Flora Mula, Virgílio António, Inocêncio Chongo, John Oludele, Kerstin Falk, Igor A. Paploski, Mitermayer G. Reis, Uriel Kitron, Beate M. Kümmerer, Guilherme S. Ribeiro, Eduardo Samo Gudo

**Affiliations:** 1 Instituto de Saúde Coletiva, Universidade Federal da Bahia, Salvador, BA, Brazil; 2 Instituto Gonçalo Moniz, Fundação Oswaldo Cruz, Ministério da Saúde, Salvador, BA, Brazil; 3 Universidade Pedagógica de Quelimane, Zambézia, Mozambique; 4 Instituto Nacional de Saúde, Ministério da Saúde, Maputo, Mozambique; 5 Public Health Agency of Sweden, Stockholm, Sweeden; 6 Department of Microbiology, Tumor and Cell Biology, Karolinska Institutet, Stockholm, Sweden; 7 Faculdade de Medicina, Universidade Federal da Bahia, Salvador, BA, Brazil; 8 Emory University, Atlanta, GE, United States of America; 9 Institute of Virology, University of Bonn Medical Centre, Bonn, Germany; CEA, FRANCE

## Abstract

**Background:**

In January 2016, health authorities from Zambézia province, Mozambique reported the detection of some patients presenting with fever, arthralgia, and a positive result for chikungunya in an IgM-based Rapid Diagnostic Test (RDT). We initiated a study to investigate a potential chikungunya outbreak in the city of Quelimane.

**Methods/Principal findings:**

From February to June 2016, we conducted a cross-sectional study enrolling febrile patients attending five outpatient health units in Quelimane. Serum from each patient was tested for CHIKV and DENV, using IgM and IgG ELISA and qRT-PCR. Patients were also tested for malaria by RDT. Entomological surveys were performed around patients’ households, and we calculated the proportion of positive ovitraps and the egg density per trap. A total of 163 patients were recruited, of which 99 (60.7%) were female. The median age was 28 years. IgM and IgG anti-CHIKV antibodies were identified in 17 (10.4%) and 103 (63.2%) patients, respectively. Plaque reduction neutralization assay confirmed the presence of anti-CHIKV antibodies in a subset of 11 tested patients with positive IgG results. IgM anti-DENV antibodies were found in 1 (0.9%) of 104 tested patients. Malaria was diagnosed in 35 (21.5%) patients, 2 of whom were also IgM-positive for CHIKV. Older age and lower education level were independently associated with the prevalence of IgG anti-CHIKV antibodies. Immature forms of *Aedes aegypti* were collected in 16 (20.3%) of 79 surveyed households. We also found that 25.0% (16/64) of the traps were positive, with an average of 90.8 eggs per pallet.

**Conclusions:**

Our investigation demonstrated that no CHIKV outbreak was ongoing in Quelimane; rather, endemic transmission of the virus has been ongoing. *Aedes aegypti* mosquitoes are abundant, but dengue cases occurred only sporadically. Further population-based cohort studies are needed to improve our understanding of aspects related to the dynamics of arboviral transmission in Mozambique, as well as in other parts of Sub-Saharan Africa.

## Introduction

Chikungunya (CHKV), Dengue (DENV) and Zika (ZIKV) are rapidly spreading worldwide, following the distribution of their shared main vectors, *Aedes aegypti* and *Aedes albopictus* mosquitoes [1–3]. The explosive spread of ZIKV in South America in 2015/2016 led the World Health Organization (WHO) to declare ZIKV as a public health emergence of international concern on February 2016 [4]. DENV and CHIKV also continue to expand worldwide, with massive outbreaks reported in several regions in recent years [5]. If we consider that the main drivers for the spread of arboviruses are the rapid and unplanned urbanization, and intensification of international trade and travel, it is expected that geographical spread of these (and other) arbovirus will continue [6].

Although Sub-Saharan Africa is the known place of origin of several arboviruses, such as ZIKV and CHIKV, which were first described in this region [7,8], and has the tropical climate favorable to the presence of *Aedes spp*. mosquitoes, studies on arboviruses in Africa are still scarce [9–11]. In Mozambique, the first dengue outbreak was identified in 1984 [12]. After a long period with no reports of dengue cases in the country, in 2013 and 2014, the Ministry of Health confirmed a new dengue outbreak, and surveillance for acute febrile illness suggested that dengue has become endemic in the northern region of the country [13,14]. In addition, in 2013, CHIKV infections were detected among febrile patients in southern Mozambique [15]. With regard to Zambézia province, no study on chikungunya has been conducted since 1957, when Kokernot *et al* found serological evidence of CHIKV [16]; DENV has never been previously investigated in this province.

Information on the ecology of *Aedes spp*. mosquitoes in Mozambique is also limited. An entomological survey conducted in the 1940s, found *Aedes spp*. mosquitoes in several parts of the country [17]. More recently, during the 2013/2014 dengue outbreak, a preliminary entomological investigation found that *Aedes aegypti* was abundant in three northern cities in Mozambique [18], and, in 2015, *Aedes albopictus* was detected for the first time in the country [19].

Since the last outbreak of dengue in Mozambique, the Ministry of Health (MoH) made efforts to expand surveillance systems for acute febrile illnesses throughout the country. In early 2016, the activities were implemented in Quelimane, a city located in Zambézia province, by the local health authorities. They introduced the Rapid Diagnostic Test (Alere Inc., Waltham, USA) for CHIKV and during the surveillance process they reported the detection of some patients presenting with fever, arthralgia, and a positive result for chikungunya. Consequently, the Mozambique National Institute of Health initiated an investigation to determine whether a chikungunya outbreak was ongoing in Quelimane.

## Methods

### Study design and site

From February 24 to June 20, 2016, we conducted a cross-sectional study to enroll patients with acute febrile illness attending outpatient clinics at five health units in Quelimane (Coalane Health Unit, 4 de Dezembro Health Unit, 24 de Julho Health Unit, 17 de Setembro Health Unit, and Quelimane Provincial Hospital). Quelimane covers an area of 117 km^2^ with an estimated population of 245,886 inhabitants [20]. The city is located by the Bons Sinais River, 20 km from the Indian Ocean, and the river port is a major contributor to the local economy. Other economic activities include family farming and artisanal fishing. About 50% of Quelimane’s population lives on less than the international indicator of poverty of US$2/day [21], in suburban neighborhoods that lack potable water supply and sanitation infrastructure. The local climate is tropical and humid with two distinct seasons, a rainy season from November through April and a dry season from May through October. The average annual precipitation is 1,395 mm and the average annual temperature is 25.7°C [22].

### Inclusion criteria and enrollment

The following inclusion criteria were used: age 5 years or older, with reported or measured fever (axillary temperature ≥ 37.5° C) for up to seven days, and presence of one or more of the following symptoms: headache, arthralgia, myalgia, retro-orbital pain and rash. Patients with a readily identifiable focus of infectious, such as pneumonia detected by chest radiography, or with suspected tuberculosis, bronchitis, pharyngitis, and cellulitis were excluded. Patients suspected to have malaria were not excluded from the study.

The investigation started in Coalane Health Unit in February 24, and from May 21 until June 20, the Ministry of Health decided to expand the investigation to the other four health units as part of the investigation of a potential outbreak. At Coalane Health Unit all patients who attended the clinic during Monday-Friday, between 7h30 and 13h00, and who fulfilled the inclusion criteria were invited to participate in the investigation. At the other health units, patients fulfilling the inclusion criteria were recruited in a non-random, non-systematic manner (e.g. the same team visited the four health units on different days and hours for patient enrollment).

### Questionnaire

At study enrollment, we interviewed the patients to collect clinical and demographic data using a structured questionnaire. We provided an oral translation of the questionnaires into the local dialect for patients who did not speak Portuguese.

### Laboratory diagnosis

All patients were initially tested for malaria using a rapid diagnostic test (RDT) specific for *Plasmodium falciparum*, according to the manufacturer's instructions (SD BIOLINE Malaria Ag P.f, SD. Inc.Yongin-si, Gyeonggi-do, South Korea). In order to investigate an arboviral infection, blood samples were collected at enrollment and centrifuged to separate the sera. Sera were kept refrigerated and then stored in the same day at -20°C in the laboratory of Quelimane Provincial Hospital until shipment to the Virus Isolation Laboratory (VIL) of the National Institute of Health, in Maputo, where serological and molecular testing of CHIKV and DENV were performed.

Commercial Enzyme Linked Immunosorbent Assays (ELISA) were used to investigate the presence of CHIKV IgM and IgG antibodies (EUROIMMUN, Lübeck, Germany), as well as DENV nonstructural protein 1 (NS1), and DENV IgM and IgG antibodies (Alere Inc., Waltham, USA). All tests were performed following manufacturer's instructions. Serum samples were also tested by real-time reverse transcriptase polymerase chain reaction (qRT-PCR) for simultaneous identification of CHIKV, DENV and ZIKV (Trioplex, Centers for Disease Control and Prevention, Atlanta, USA) [23]. Positive control was used for both ELISA and qRT-PCR reactions. In order to increase the confidence in the CHIKV IgG ELISA results, a subset of 12 samples, of which 11 were IgG positive for CHIKV, was sent to the Institute of Virology, University of Bonn Medical Centre, Bonn, Germany to be tested for CHIKV by plaque reduction neutralization test (PRNT), using the Virus Replicon Particles (VRP) neutralization assay. VRP was performed on BHK-21 cells as previously described [24].

### Case definitions

We defined a case of acute arboviral infection as a patient with a positive qRT-PCR test result for DENV or CHIKV. A patient with a positive NS1 ELISA result was also considered as a case of acute DENV infection. Patients in which we detected IgM antibodies to DENV or CHIKV, regardless of the presence of IgG antibodies, were defined as cases of recent arboviral infection. Patients in which we detected IgG antibodies to DENV or CHIKV in absence of IgM antibodies were defined as cases of previous arboviral infection. Patients with negative results for all tests were considered negative for DENV or CHIKV. Patients presenting a positive RDT for malaria were defined as having *Plasmodium falciparum* malaria.

### Entomological assessment

Entomological surveys were carried out in the households of the study patients who were recruited at the Coalane Health Unit, within five days of study enrollment. At each household, we conducted inspections for the presence of water-holding containers inside and outside the houses, and collected mosquito immatures from accumulated water, whenever they were present. One ovitrap was placed at each household to detect oviposition and for collection of immature *Aedes* mosquitoes. All larvae (from breeding sites or ovitraps) were reared at 27 ± 2°C for up to 10 days at the provincial laboratory of entomology in Quelimane, until they emerged. Then, the adult mosquitoes were killed in a cold chamber for further identification and classification of species. The geographical coordinates of each household were obtained using a Garmin Trex portable GPS (Global Positioning System).

### Statistical analysis

Data were entered into Excel 2013 (Microsoft Inc, Redmond, USA) and REDCap (Research electronic data capture–Vanderbilt University, Nashville, USA) [25] databases, and analyzed using STATA 12 statistical software package (StataCorp LP, College Station, USA). We calculated medians and interquartile ranges for quantitative variables and absolute and relative frequencies for categorical variables. Kruskal Wallis, Pearson Chi-square or Fisher exact test were used as appropriate. We set the two-tail significance level at P<0.05. Associations between sociodemographic factors and prevalence of CHIKV IgG antibodies were assessed using bivariate and multivariate log-binomial regression analyses. For this analysis, age was stratified into three groups: 5–19, 20–39, and ≥40 years. Associations between sociodemographic characteristics and other test results (for DENV and CHIKV IgM) were not assessed due to the low frequency of positive results.

We also calculated the proportion of positive ovitraps and the average number of eggs per positive trap. Geographical coordinates of patient’s households were entered in Qgis 2.14 software (Open Source Geospatial Foundation—OSGeo, Beaverton, USA), within the *openstreetmap* database (OpenStreetMap Foundation, Sutton Coldfield, United Kingdom), and analyzed to illustrate the spatial distribution of the surveyed households. We used Moran's I index to measure spatial autocorrelation.

### Ethics statement

This study was conducted as part of an investigation of a potential chikungunya outbreak after few patients tested positive for CHIKV using RDT in Coalane Health Unit. Local authorities contacted National Institute of Health to investigate. Since this was part of an investigation of potential outbreak, all participants provided only verbal consent. We only requested verbal consent because this surveillance was embedded into routine care, and we did not want to change the routine assistance being provided to these patients. Patients under 18 years old were recruited after verbal consent was provided by their parents, followed by their verbal assent.

## Results

### Socio-demographics characteristics of the participants

We enrolled 163 acute febrile illness patients. Median age was 28 years (interquartile rage: 20–40 years), all patients were black, 99 (60.7%) were female, 41 (25.2%) were illiterate, and 94 (64.4% of the 146 aged ≥18 years) were unemployed. No patient had a history of travel to countries where the studied arboviruses are considered endemic. All patients lived in Quelimane, although some worked in nearby districts.

### Frequency of chikungunya and dengue

**Fig 1:**
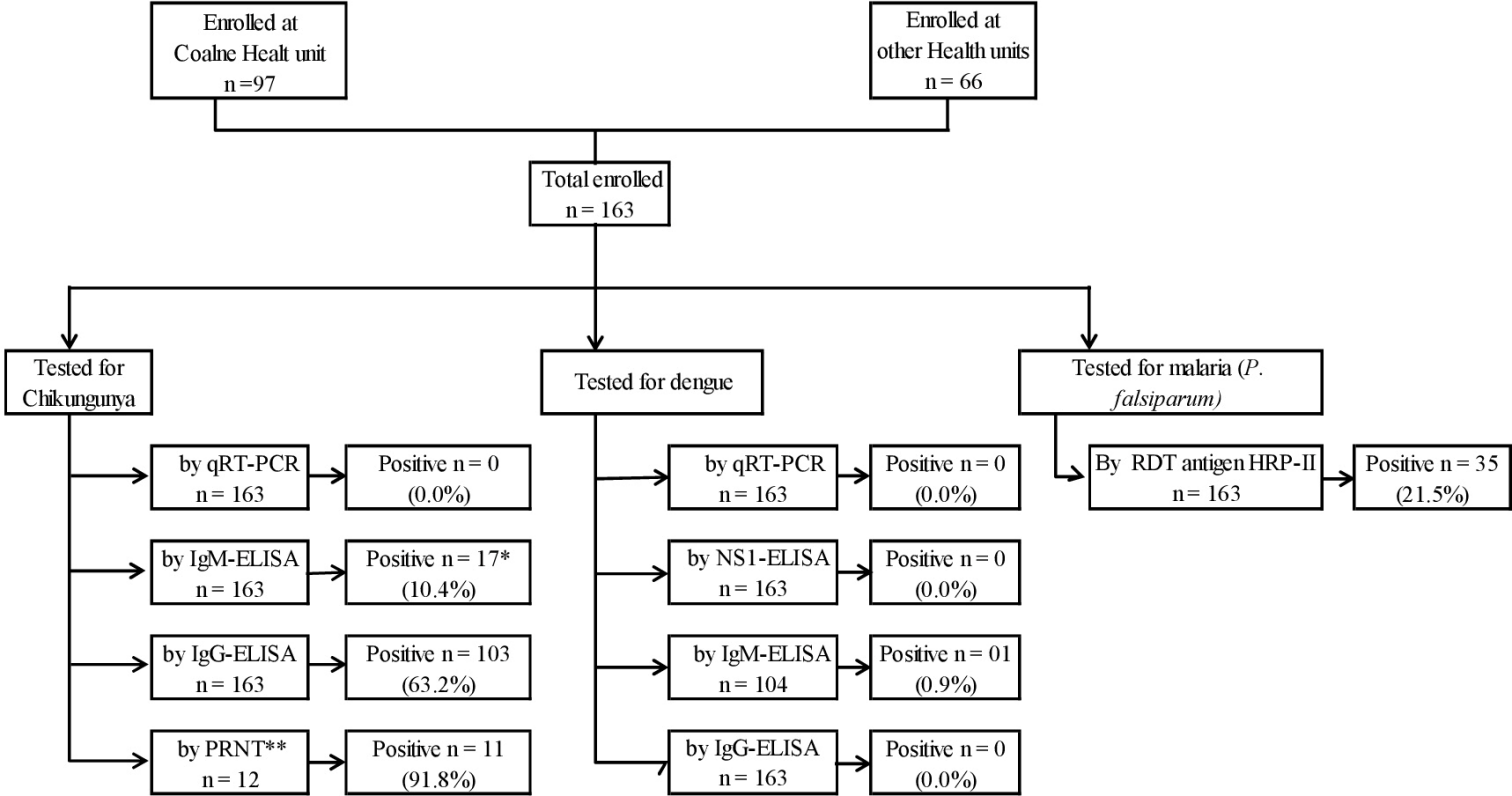
Diagram of enrollment of study patients and performed tests. * Out of 17 patients who had a positive IgM-ELISA for CHIKV, 2 (11.8%) also had a positive malaria rapid diagnostic test. ** Of the 12 samples shipped to neutralization, 11 were chosen from the 103 IgG anti-CHIKV positive by ELISA.

All of the 163 patients were qRT-PCR negative for chikungunya (Fig 1). IgM and IgG anti-CHIKV antibodies were detected in 17 (10.4%) and 103 (63.2%) patients, respectively (12 [7.4%] patients were positive for both IgM and IgG). From the subset of 12 samples tested later by PRNT, all 11 IgG positive samples were also PRNT-positive, with titers ranging from 100 to 3,200. The only sample tested by PRNT that was IgG negative for CHIKV was also negative by PRNT.

According to our case definition for chikungunya, 17 (10.4%) patients had a recent CHIKV infection, 91 (55.8%) had a previous CHIKV infection, and 55 (33.7%) had never been infected by CHIKV. Patients diagnosed with a recent or previous CHIKV infection were older and had a lower education level than those who were negative for CHIKV (P<0.01) (Table 1). The only statistically significant clinical distinction between the patients was the presence of chills, which were more common among those negative for CHIKV (92.7%) than among those with a recent (82.4%) or a previous (73.6%) CHIKV infection (P<0.01) (Table 1). Patients with laboratory evidence of chikungunya infection were detected in all health centers, except at the provincial hospital.

**Table 1:** Demographic and clinical characteristics of study participants stratified by case definition to CHIKV infection.

Characteristics	Recent infection^a^	Previous infection^b^	Negative^c^	p-value
	n (%) or median (interquartile range)			
**Total**	17 (10.4)	91 (55.8)	55 (33.7)	
**Demographics**				
**Age**	29 (16–35)	33 (24–45)	22 (19–29)	<0.01
**Males**	9 (52.9)	31 (34.1)	24 (43.6)	0.25
**Education level**				<0.01
Illiterate	6 (35.3)	30 (33.0)	5 (9.1)	
Primary	5 (29.4)	27 (29.6)	8 (14.5)	
Secondary or University	6 (35.3)	34 (37.4)	42 (76.4)	
**Work**^d^	5 (41.8)	29 (33.0)	18 (39.1)	0.70
**Clinical**				
Days since fever onset	4 (2–6)	2 (1–3)	2 (1–3)	0.13
Headache	17 (100)	85 (93.4)	52 (94.6)	0.88
Arthralgia	15 (88.2)	80 (87.9)	49 (89.1)	1.00
Chills	14 (82.4)	67 (73.6)	51 (92.7)	0.01
Myalgia	11 (64.7)	70 (76.9)	37 (67.3)	0.32
Weakness	13 (76.5)	58 (63.7)	35 (63.6)	0.66
Anorexia	8 (47.1)	42 (46.2)	29 (52.7)	0.74
Abdominal pain	6 (35.3)	30 (33.0)	20 (36.4)	0.91
Retro orbital pain	7 (41.2)	31 (34.1)	11 (20.0)	0.11
Vomiting	1 (5.9)	11 (12.1)	6 (10.9)	0.94
Diarrhea	2 (11.8)	6 (6.7)	6 (10.9)	0.51

^**a**^A case of recent CHIKV infection was defined by detection in the ELISA of IgM anti-CHIKV antibodies in a patient with a negative qRT-PCR test, regardless of the ELISA result for the IgG anti-CHIKV.

^**b**^A case of previous CHIKV infection was defined by detection in the ELISA of IgG anti-CHIKV antibodies, in a patient with negative results in the qRT-PCR and IgM ELISA for CHIKV.

^**c**^A case negative for CHIKV infection was defined by CHIKV negative results in either qRT-PCR, IgM and IgG ELISA

^**d**^Data was only analyzed for the 146 patients aged ≥ 18 years: 12 patients with a recent infection, 88 patients with previous CHIKV infection, and 46 patients who tested negative for CHIKV.

All of the 163 patients tested negative for DENV by qRT-PCR, NS1-ELISA or IgG-ELISA, though one (0.9%) of 104 patients tested by DENV IgM-ELISA was positive (Fig 1). Malaria was detected in 35 (21.5% of 163) patients, and 2 of them were also positive for CHIKV by IgM-ELISA. None of the laboratory-confirmed patients presented severe disease requiring special care or hospitalization.

### Factors associated with CHIKV infection

Age group and educational level were associated with previous CHIKV infection in both bivariate and multivariate analyses (Table 2). In the multivariate analysis, the adjusted prevalence ratios for the age groups 20–39 and ≥40 years, in comparison to the 5–19 years group, were 1.55 (95% confidence interval [CI]: 1.02–2.34) and 1.89 (95% CI: 1.28–2.83), respectively. The adjusted prevalence ratios of previous CHIKV infection for those with any primary level education and for illiterate patients were 1.75 (95% CI: 1.36–2.25) and 1.48 (95% CI: 1.12–1.94), respectively, compared to those with at least some secondary education.

**Table 2:** Prevalence of IgG anti-CHIKV antibodies according to sociodemographic factors (N = 163).

Variables		Total	CHIKV IgG positive, n (%)	Crude		Adjusted	
				PR	95% CI	PR	95% CI
**Sex**							
	Female	99	65 (65.7)	1.00			
	Male	64	38 (59.4)	0.90	0.71;1.15		
**Age group (years)**							
	5–19	32	14 (43.8)	1.00		1.00	
	20–39	90	54 (60.0)	1.37	0.89;2.10	1.55	1.02;2.34
	≥40	41	35 (85.4)	1.95	1.29;2.95	1.89	1.28;2.83
**Employment**^a^							
	No	94	64 (68.1)	1.00			
	Yes	52	34 (65.4)	0.96	0.75;1.22		
**Education level**							
	Secondary or University	82	39 (47.6)	1.00		1.00	
	Primary	40	32 (80.0)	1.68	1.28;2.21	1.75	1.36;2.25
	Illiterates	41	32 (78.1)	1.64	1.24;2.17	1.48	1.12;1.94

PR = Prevalence ratio; 95% CI = 95% confidence interval.

^**a**^Only for patients aged ≥ 18 years (N = 146)

### Entomological inspection for *Aedes spp*.

**Fig 2:**
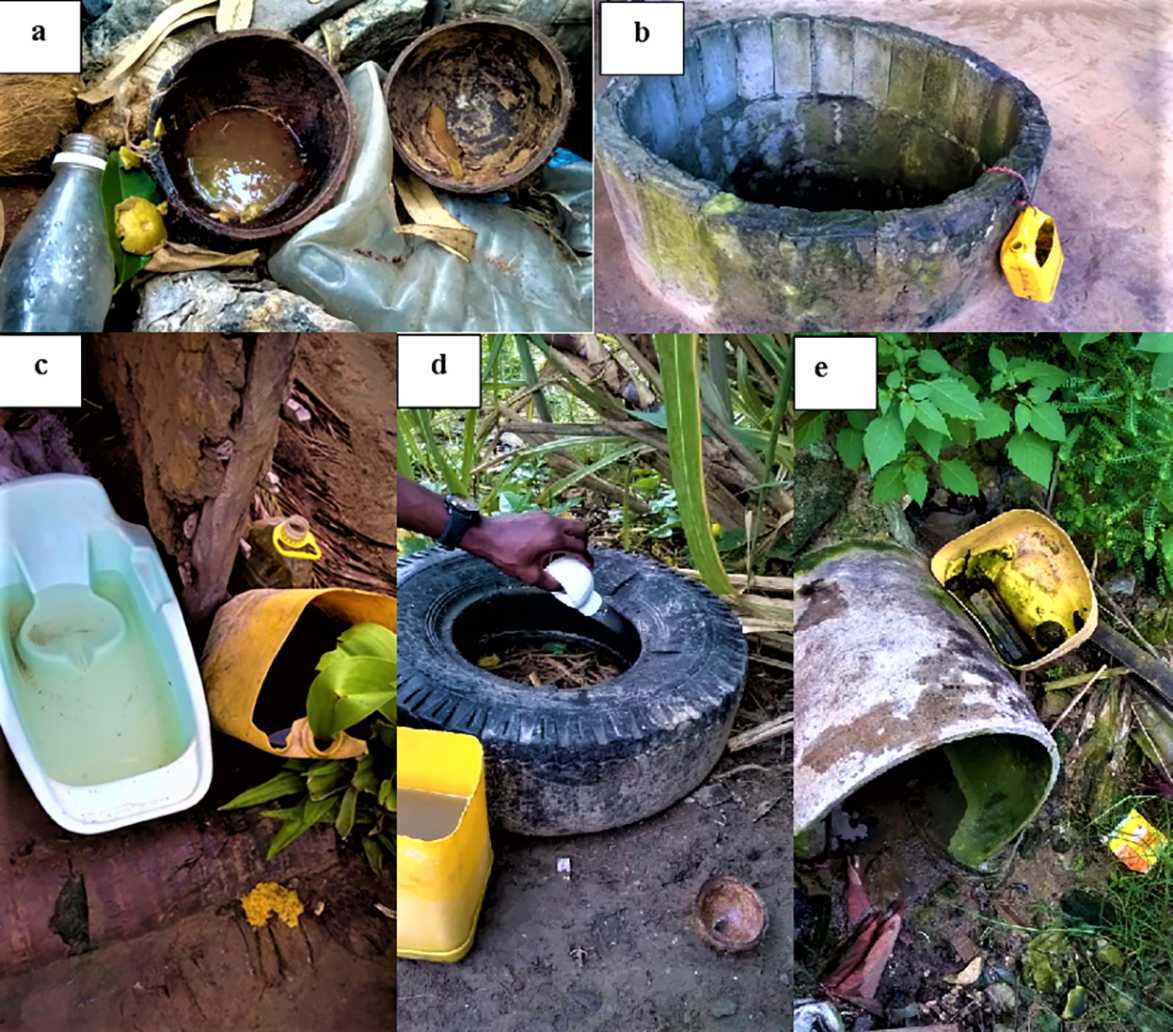
Different water containers serving as breeding sites for *Aedes aegypti* mosquitoes around the patients’ households. a) Coconut shell; b) unclosed well; c) basin and buckets; d) tire; e) sewer pipe.

**Fig 3:**
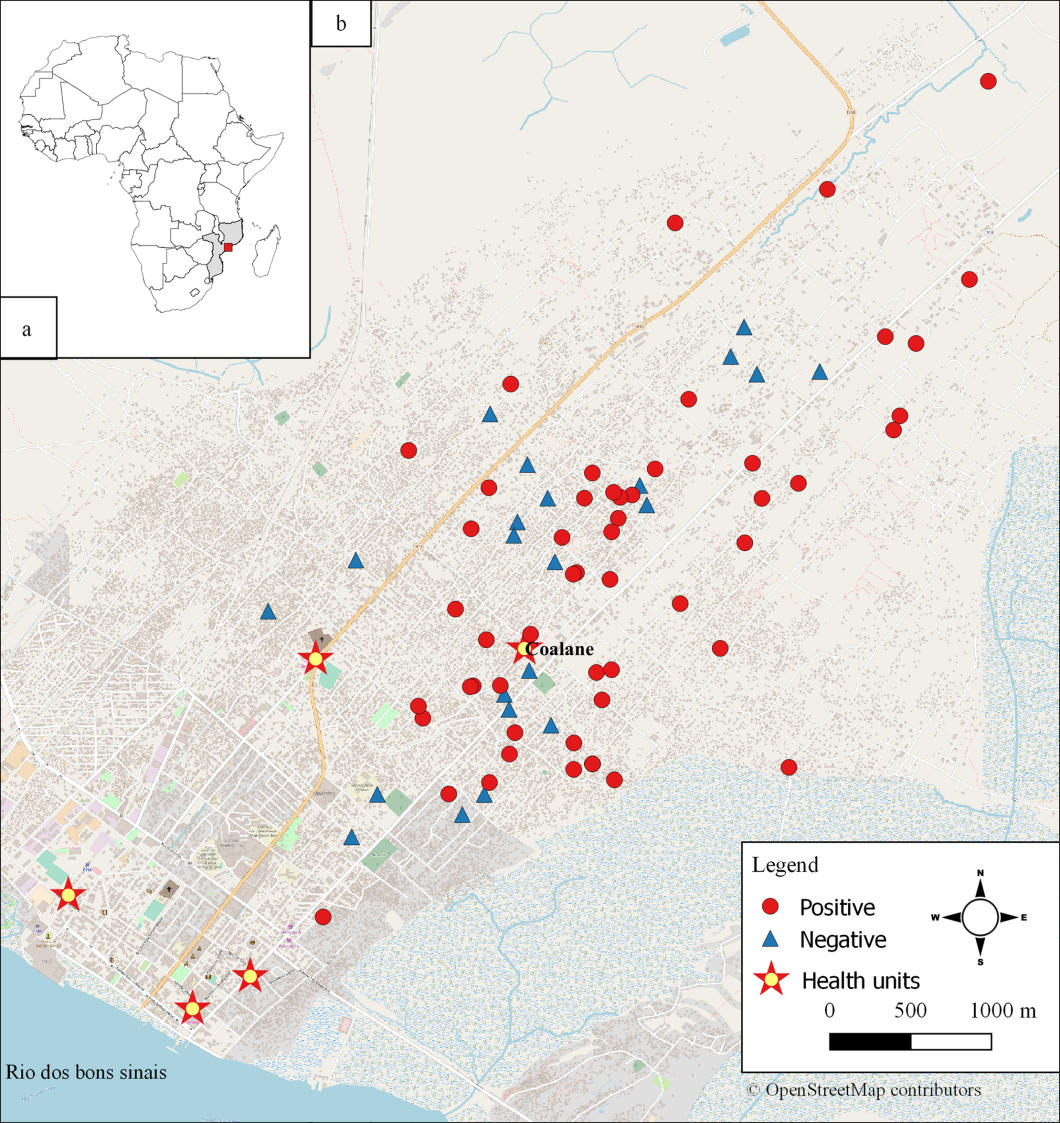
Spatial distribution of the households of the patients recruited at the Coalane health units. A: geographical location of Mozambique on the East coast of Africa and Quelimane in the central region of the country; B: map of Quelimane. In red are the households of the patients who had anti-CHIKV IgM, IgG, or both antibodies in the serum; and in blue, the households of the patients whose serum was negative for chikungunya.

We were able to survey the household of 79 study patients, of which 57 (72.2%) had a laboratory evidence of previous CHIKV infection. *Aedes* larvae were found in 16 (20.3%) households. The breeding sites were milk cans (n = 4), wells, buckets, coconut shells, tires, and washing tanks (2 of each), sewage pipe, drum, pan, and landfill (1 of each) (some households presented more than one breeding site) (Fig 2). Ovitraps were placed in 64 of the 79 surveyed households and eggs were found in 16 (25.0%) of them. The mean number of eggs per pallet in the 16 ovitraps with eggs was 90.8. A total of 204 adult *Aedes* mosquitoes that emerged from larvae collected in breeding sites and ovitraps were further identified, all of them as *Aedes aegypti*. Among them, 78 (38.2%) were males and 126 (61.8%) were females. Non-*Aedes* mosquitoes were discarded. The spatial distribution of the surveyed households is depicted in Fig 3. No clustering pattern of the households of patients with positive chikungunya results was observed within a radius of 100 meters between neighbors (Moran I value of 0.37; IC95%: -0.84; 1.58).

## Discussion

Our findings suggest that CHIKV infections are common, while DENV infections occurs sporadically among febrile patients in Quelimane. In addition, we found anti-CHIKV IgG antibodies in more than 60% of the patients, and observed that older patients had higher antibody positivity, supporting the hypothesis that the virus has been silently circulating in the region for decades. Therefore, the investigation point to an endemic transmission of CHIKV in the city and suggest that there was no ongoing outbreak.

Because all qRT-PCR results for CHIKV were negative, it is possible that other *Alphavirus* circulating in Quelimane were causing infections that cross-react with CHIKV serologically. However, the test-to-test comparison between the results of the IgG-ELISA and the neutralization assay showed 100% agreement, supporting CHIKV as the cause of the detected infections. The presence of CHIKV in Quelimane was not surprising, because chikungunya has been reported in neighboring countries [26,27] and the prevalence of previous CHIKV infections in East Africa has been estimated as 72% in Kenya in 2004 [28] and 45% in Madagascar in 2009 [29]. In Mozambique, serological surveys first detected evidence of CHIKV infections in 1957 [16]. More recently, cases of CHIKV were confirmed in Maputo [15], the capital of Mozambique, ~1,600 km south of Quelimane, in 2013, and in Cabo Delgado [30], northern Mozambique, in 2014 [30].

The frequency of 63.2% of CHIKV previous infections in our patient sample is the highest ever reported in Mozambique. Of note, previous CHIKV infection was more common among those with lower education level. Assuming that educational level is a proxy for socioeconomic status, this finding suggests that poor living conditions may be a risk factor for CHIKV infections, as also reported in studies in Indian Ocean islands [31] and in Malaysia [32].

The spatial distribution of the household addresses of the patients enrolled at the Coalane Health unit provided an idea of how diffuse CHIKV occurrence was in Quelimane. We found cases in neighborhoods as far as 7.5 km from each other. We noted that most of the patients with laboratory evidence of CHIKV infection resided close to the Coalane Health Unit. Clustering of arboviral cases (and of other infections) near health units has been previously documented, and can be explained by ease of access [33]. It is likely that had we been able to geocode the home address of patients enrolled in the other health units, we would have observed the same pattern of case clustering around them.

Only one febrile patient presented IgM anti-DENV antibodies, and none was positive by IgG-ELISA. If the low frequency of DENV infections holds for the citywide population, we can assume that DENV transmission has been very low in Quelimane, and that the population is largely susceptible. This is in contrast to reports from northern Mozambique [13] where a high frequency of DENV infections was recently recognized.

In one of five households investigated, immature forms of *Ae*. *Aegypti* were found. There is no prior study in Mozambique investigating the proportion of households containing immature *Aedes spp*., but surveys performed using tire traps in the capital Maputo and in the northern cities of Pemba, Nampula and Nacala found *Ae*. *aegypti* larvae in 19,3% (16/83), 72.0% (18/25), 59.0% (23/39), and 64.5% (20/31), respectively [18]. Altogether, these observations suggest that this mosquito vector is widely spread throughout Mozambique.

Given the likely low level of DENV immunity in the Quelimane population, the high abundance of *Ae*. *aegypti* mosquitoes in the city, and the circulation of the virus in other Mozambican cities, there is potential for dengue outbreaks in this region of the country, as well as for other arboviruses for which *Ae*. *aegypti* is a competent vector, such as ZIKV and yellow fever virus. The emergence of DENV and other arboviruses may also be facilitated by the large variety of *Ae*. *aegypti* breeding sites, coupled with the poor local sanitation infrastructure.

All study patients were screened for *P*. *falciparum* infections and 21.5% tested positive for malaria. The most recent population-based malaria survey conducted in Mozambique found 67.6% prevalence of *Plasmodium falciparum* infections in children from zero to five years of age from Zambézia province (data from the Mozambique National Institute of Health, 2015) [34] and in different regions of the neighboring country Tanzania, the frequency of malaria among febrile patients varied between 10.8% and 46.9%, between 2010 and 2012 [35]. Thus, in such places where malaria is hyperendemic and CHIKV transmission is occurring simultaneously, malaria and CHIKV co-infections are likely to occur, increasing the diagnostic challenge, and, consequently, the proper clinical management. Investment in laboratory capacity will be necessary to aid in the differential diagnosis of acute febrile patients.

Our study has several limitations. First, more than half of our patients were enrolled in one of the five study health units. However, we detected patients with laboratory evidence of CHIKV infection in four of the health units, suggesting that CHIKV transmission occurs throughout Quelimane. Second, we were not able to collect convalescent-phase serum, hampering detection of seroconversions and serological diagnosis of patients presenting soon after disease onset. Third, serum samples were stored at -20 ^o^C for 30–45 days in Quelimane before being sent to Maputo in order to perform the qRT-PCR tests, and this may have reduced our ability to detect viral RNA. Lastly, although we have detected abundant *Ae*. *aegypti* infestation near the study participants’ households, we did not capture adult specimens to verify whether they were infected by CHIKV.

## Conclusion

We conclude that no CHIKV outbreak was ongoing in Quelimane; rather, the new diagnostic routine implemented during acute febrile illnesses surveillance revealed that the virus has been endemically transmitted without detection by health professionals and public health authorities. Implementing laboratory tools will be critical to ensure proper differentiation between arboviral infections, malaria, and other febrile illness. *Ae*. *aegypti* mosquitoes are abundant in the region, and likely serve as the main vector for CHIKV transmission. Their abundance also poses the population to risk for arboviral outbreaks, especially DENV, for which the population likely has a low level of immunity. Further population-based cohort studies are needed to improve understanding of aspects related to the dynamics of arboviral transmission in Mozambique and Sub-Saharan Africa.

## Supporting information

S1 Text STROBE statement for observational studies checklist. (PDF)

S1 Dataset Clinical, epidemiological and laboratory data of the patients recruited at health units. (XLSX)

S2 Dataset Mosquitoes classification. (XLSX)

S1 Table Dictionary of variables of S1 Dataset. (XLSX)
