# Supplementary material for: Evidence for chikungunya and dengue transmission in Quelimane, Mozambique: Results from an investigation of a potential outbreak of chikungunya virus
Source: PLoS One. 2018 Feb 7;13(2):e0192110. doi: 10.1371/journal.pone.0192110 (PMC5802900; doi:10.1371/journal.pone.0192110)
Supplement: S1 Text — (PDF) [file pone.0192110.s001.pdf]

STROBE Statement—checklist of items that should be included in reports of observational studies

	Item No.	Recommendation	Page No.	Relevant text from manuscript
**Title and abstract**	1	(*a*) Indicate the study's design with a commonly used term in the title or the abstract	**[pages,2]**	**Within the method section of the abstract**
		(*b*) Provide in the abstract an informative and balanced summary of what was done and what was found	**[page 2]**	**See results section of abstract**
**Introduction**				
Background/rationale	2	Explain the scientific background and rationale for the investigation being reported	**[page 3-4]**	
Objectives	3	State specific objectives, including any prespecified hypotheses	**[page 4]**	**Paragraph 3**
**Methods**				
Study design	4	Present key elements of study design early in the paper	**[page 5]**	**Methods, paragraph 1**
Setting	5	Describe the setting, locations, and relevant dates, including periods of recruitment, exposure, follow-up, and data collection	**[pages 5-7]**	**Methods**
Participants	6	(*a*) *Cohort study*—Give the eligibility criteria, and the sources and methods of selection of participants. Describe methods of follow-up	N/A	
		Case-control study—Give the eligibility criteria, and the sources and methods of case ascertainment and control selection. Give the rationale for the choice of cases and controls	N/A	**Section of the inclusion criteria and enrollment and section of questionnaire**
		Cross-sectional study—Give the eligibility criteria, and the sources and methods of selection of participants	**[pages 5-6]**	
		(*b*) *Cohort study*—For matched studies, give matching criteria and number of exposed and unexposed	**N/A**	
		Case-control study—For matched studies, give matching criteria and the number of controls per case	**N/A**	
Variables	7	Clearly define all outcomes, exposures, predictors, potential confounders, and effect modifiers. Give diagnostic criteria, if applicable	**[pages 5-7]**	**Section of the case definitions, section of inclusion criteria and enrollment and section of questionnaire**
Data sources/	8*	For each variable of interest, give sources of data and details of methods of assessment	**[pages 5 and**	**Section of inclusion criteria**

measurement		(measurement). Describe comparability of assessment methods if there is more than one group	page 8]	**and enrollment; section of questionnaire and section of the statistical analysis**
Bias	9	Describe any efforts to address potential sources of bias	**[page 5-6]**	**Section of inclusion criteria and enrollment**
Study size	10	Explain how the study size was arrived at	**[page 5-6]**	**Section of inclusion criteria and enrollment paragraph 2**
Quantitative variables	11	Explain how quantitative variables were handled in the analyses. If applicable, describe which groupings were chosen and why	**[page 8]**	**Section of the statistical analysis paragraph 1**
Statistical methods	12	(*a*) Describe all statistical methods, including those used to control for confounding	**[pages 8]**	**Section of the statistical analysis paragraph 1**
		(*b*) Describe any methods used to examine subgroups and interactions	**[N/A]**	
		(*c*) Explain how missing data were addressed	**[none]**	
		(*d*) *Cohort study*—If applicable, explain how loss to follow-up was addressed<br>*Case-control study*—If applicable, explain how matching of cases and controls was addressed<br>*Cross-sectional study*—If applicable, describe analytical methods taking account of sampling strategy	**N/A**<br><br>**N/A**	
		(*e*) Describe any sensitivity analyses	**N/A**	
**Results**				
Participants	13*	(a) Report numbers of individuals at each stage of study—eg numbers potentially eligible, examined for eligibility, confirmed eligible, included in the study, completing follow-up, and analysed	**Fig 1**	
		(b) Give reasons for non-participation at each stage	**N/A**	
		(c) Consider use of a flow diagram	**Fig 1**	
Descriptive data	14*	(a) Give characteristics of study participants (eg demographic, clinical, social) and information on exposures and potential confounders	**[Results page 9 and page 10]**	**Section of the socio-demographics characteristics of the participants; table 1**
		(b) Indicate number of participants with missing data for each variable of interest	**[none]**	
		(c) *Cohort study*—Summarise follow-up time (eg, average and total amount)		
Outcome data	15*	*Cohort study*—Report numbers of outcome events or summary measures over time	**N/A**	
		Case-control study—Report numbers in each exposure category, or summary measures of exposure	**N/A**	

		Cross-sectional study—Report numbers of outcome events or summary measures	**[page 9-11]**	**Section of the frequency of chikungunya and dengue paragraph 1-3**
Main results	16	(*a*) Give unadjusted estimates and, if applicable, confounder-adjusted estimates and their precision (eg, 95% confidence interval). Make clear which confounders were adjusted for and why they were included	**[page 11 - 12]**	**Section of the factors associated with CHIKV infection; table 2**
		(*b*) Report category boundaries when continuous variables were categorized	**[page 11]**	**Section of the factors associated with CHIKV infection**
		(*c*) If relevant, consider translating estimates of relative risk into absolute risk for a meaningful time period	**N/A**	
Other analyses	17	Report other analyses done—eg analyses of subgroups and interactions, and sensitivity analyses	**N/A**	
**Discussion**				
Key results	18	Summarise key results with reference to study objectives	**[page 9 and page 12]**	**Section of the Frequency of chikungunya and dengue; section of the entomological inspection for *Aedes spp***
Limitations	19	Discuss limitations of the study, taking into account sources of potential bias or imprecision. Discuss both direction and magnitude of any potential bias	**[page15]**	**Paragraph 3**
Interpretation	20	Give a cautious overall interpretation of results considering objectives, limitations, multiplicity of analyses, results from similar studies, and other relevant evidence	**[page 12-15]**	**Discussion**
Generalisability	21	Discuss the generalisability (external validity) of the study results	**[page 14, 16]**	**Discussion paragraph 3,4 and conclusion**
**Other information**				
Funding	22	Give the source of funding and the role of the funders for the present study and, if applicable, for the original study on which the present article is based	**[entered in the financial disclosure section of the submission system]**	

*Give information separately for cases and controls in case-control studies and, if applicable, for exposed and unexposed groups in cohort and cross-sectional studies.

Note: An Explanation and Elaboration article discusses each checklist item and gives methodological background and published examples of transparent reporting. The STROBE checklist is best used in conjunction with this article (freely available on the Web sites of PLoS Medicine at http://www.plosmedicine.org/, Annals of Internal Medicine at http://www.annals.org/, and Epidemiology at http://www.epidem.com/). Information on the STROBE Initiative is available at www.strobe-statement.org.
